# Prevalence of Positive Diabetes-Associated Autoantibodies among Type 2 Diabetes and Related Metabolic and Inflammatory Differences in a Sample of the Bulgarian Population

**DOI:** 10.1155/2017/9016148

**Published:** 2017-05-09

**Authors:** Emanuela Tsvetkova Zaharieva, Tsvetelina Veselinova Velikova, Adelina Dimitrova Tsakova, Zdravko Asenov Kamenov

**Affiliations:** ^1^University Hospital Alexandrovska, Clinic of Endocrinology, Department of Internal Medicine, Faculty of Medicine, Medical University-Sofia, Sofia, Bulgaria; ^2^University Hospital St. Ivan Rilski, Laboratory of Clinical Immunology, Department of Clinical Immunology, Faculty of Medicine, Medical University-Sofia, Sofia, Bulgaria; ^3^University Hospital Alexandrovska, Central Clinical Laboratory, Department of Clinical Laboratory, Faculty of Medicine, Medical University-Sofia, Sofia, Bulgaria

## Abstract

**Background:**

The study aimed to estimate the prevalence of unrecognized cases with positive autoantibodies among type 2 diabetes (T2D) in a sample of the Bulgarian population and to compare some metabolic and inflammatory markers to those of patients having negative autoantibodies and subjects with latent autoimmune diabetes (LADA).

**Methods:**

Patients with T2D, patients with LADA, and control participants were enrolled. Antiglutamic acid decarboxylase, anti-insulinoma-associated 2, and antizinc transporter 8 autoantibodies were assayed through ELISA. C-reactive protein and interleukin 6 (IL-6) and tumor necrosis factor alpha were assessed.

**Results:**

Ten percent of patients with T2D had positive autoantibodies. They had lower body mass index (*p* = 0.014), worse glycemic control (HbA1c, *p* = 0.033), and better HDL cholesterol (*p* = 0.026) than those in negative autoantibodies cases. Compared to LADA, glycemia and anthropometric data did not differ significantly but metabolic syndrome was more prevalent among newly found cases with positive autoantibodies (*p* = 0.046). Their level of inflammatory markers was similar to that of patients having negative autoantibodies (*p* > 0.05), but IL-6 was higher when compared to LADA (*p* = 0.002).

**Conclusion:**

Prevalence of patients having positive autoantibodies within T2D in the analyzed sample of the Bulgarian population was 10%. They shared common metabolic features with subjects with LADA, but inflammatory phenotype was closer to that of T2D.

## 1. Introduction

Whilst type 1 diabetes mellitus (T1D) is most commonly diagnosed in children and type 2 diabetes mellitus (T2D) predominantly affects adults, it is hard to establish any age boundaries between both forms of the disease. Adult-onset autoimmune diabetes encompasses a spectrum of clinical presentations. In some cases, presentation of the disease is abrupt, with ketoacidosis, but in others, hyperglycemia can be mild and even found accidentally.

In 1995, Zimmet described a group of patients with the clinical and laboratory features of T2D, together with positive anti-islet autoantibodies. Despite the obvious autoimmune characteristics of the disease, the acute presentation of diabetes was absent and the insulin dependence occurred later than in T1D, but much earlier than in T2D. This type of diabetes has been variously described as latent autoimmune diabetes in adults (LADA), diabetes type 1.5, autoimmune diabetes in adults with slowly progressive *β*-cell failure, and so forth [1–3]. Although questioned repeatedly [4], current criteria for the diagnosis of LADA remain age at the onset, the presence of positive diabetes-associated autoantibodies—islet-cell cytoplasm autoantibodies (ICA), glutamic acid decarboxylase autoantibodies (GAD65A), insulinoma-associated-2 antibodies—tyrosine phosphatase associated (IA-2A), zinc transporter 8 autoantibodies (ZnT8A); and satisfying glycemic control without insulin treatment for at least 6 months after diagnosis [5–8]. Frequency of positive autoantibodies among patients initially diagnosed with T2D varies between 4 and 14% [9].

LADA has become the subject of genetic, immunologic, clinical studies. It has been compared with both T1D and T2D. They have common predisposition haplotypes—together with T1D—HLA, INS VNTR, PTPN22, and T2D—TCF7L2 [10]. Immunopathogenesis includes common autoantibodies for T1D and LADA, and studies concerning changes in the level of inflammatory cytokines in LADA also demonstrate similarity to those in T1D rather than T2D [11, 12].

Phenotype characteristics are usually the first indication for the need to verify autoantibody status. Patients with positive diabetes-associated autoantibodies usually have lower body mass index (BMI), better hypertensive control, and better lipid status compared to those of subjects without autoantibodies [13, 14]. The prevalence of metabolic syndrome among LADA patients is lower than that in T2D, but still exceeds that in T1D [15]. However, some studies claim a lack of difference in phenotype and even similar insulin resistance between T2D and LADA. This suggests that autoantibodies should be actively assayed to exclude autoimmune diabetes [16, 17].

The aim of the study was to analyze the prevalence of unrecognized cases with positive diabetes-associated autoantibodies among patients initially diagnosed with T2D, to describe their metabolic features and characterize the alterations in high sensitivity C-reactive protein (hs-CRP), interleukin 6 (IL-6), and tumor necrosis factor alpha (TNF*α*) in comparison with participants having negative autoantibodies and patients already known to meet the criteria for LADA.

## 2. Subjects and Methods

The study included 128 patients with T2D (70 women and 58 men), 14 participants (11 women and 3 men) with LADA, and 38 control subjects (20 women and 18 men). The protocol of the study was in accordance with the Declaration of Helsinki and was approved by the local ethics committee [18]. Informed written consent was obtained from all participants. Inclusion criteria were age between 35 and 65 years, T2D diagnosed according to the criteria of the American Diabetes Association [19]. Patients with LADA were included if they meet the following three criteria: (1) onset at the age of 35 or more; (2) presence of positive diabetes-associated autoantibodies; and (3) history of satisfying glycemic control without insulin treatment for at least 6 months after diagnosis [5]. The control group included clinically healthy volunteers with BMI < 30 kg/m^2^. Exclusion criteria were age lower than 35 or older than 65 years, T1D, concomitant decompensated endocrine disorder, acute or chronic inflammatory disease, malignancies, estimated glomerular filtration rate < 60 ml/min/1.73 m^2^, hepatic diseases with transaminase enzymes over twice the upper reference range, and psychiatric disorders.

A full medical history was taken, and a physical examination was performed, including anthropometric data (weight, height, body mass index—BMI, waist circumference—WC). All (100%) LADA patients and 39 (25%) of the patients with T2D were receiving insulin. Complete blood count, lipid and lipoprotein profile, fasting plasma glucose (FPG) and 2 hours post lunch meal postprandial plasma glucose (PPG), and HbA1c in the enrolled subjects were assessed by standard techniques in the Central Laboratory of the University hospital, which is the referent one for Bulgaria. All patients and control subjects were assayed for GAD65A and IA-2A. ZnT8A was measured in 51 cases with T2D, 7 cases with LADA and 13 control participants.

IA-2A and GAD65A were assayed by enzyme-linked immunosorbent assay (ELISA) (Euroimmune Medizinische Labordiagnostika AG, Germany). The sensitivity and specificity of assays, evaluated in the 2005 Diabetes Autoantibody Standardization Program workshop, for GAD65A was 92% and 98%, respectively, and for IA-2A—66% and 99%, respectively [20]. Diagnostic cut off was 10 IU/ml for both assays. ZnT8A was analyzed through ELISA (BioVendor – Laboratorní medicína a.s., Czech Republic) with 99% (*n* = 90) specificity and 72% (*n* = 50) sensitivity, evaluated in the Islet Autoantibodies Standardization Program 2012. Assay cut off was 15 IU/ml.

Interleukin 6 (IL-6) was analyzed by Electro-Chemiluminescence Immunoassay and hs-CRP by Particle Enhanced Immunoturbidimetric Assay, both implemented on automated analyzer Cobas Integra™ 400+ (Roche Diagnostics GmbH, Germany) with lower detection limit of 1.5 pg/ml and 0.15 mg/l, respectively. TNFα was analyzed by ELISA (Gene probe, Diaclone, France) with a sensitivity of 8 pg/ml.

### 2.1. Metabolic Syndrome

Metabolic syndrome was assessed according to the National Cholesterol Education Program (NCEP) criteria: (1) waist circumference > 102 cm in men and >88 cm in women; (2) triglycerides ≥1.70 mmol/l; (3) HDL cholesterol < 1.03 mmol/l in men and <1.29 mmol/l in women or receiving lipid lowering therapy; (4) blood pressure ≥ 130/85 mmHg or taking antihypertensive medication; and (5) fasting glucose criterion was considered positive for all patients with diabetes [21].

### 2.2. Statistical Analysis

Statistical analysis was performed with SPSS 19 Statistics Package (Chicago, Illinois). The difference between independent samples was estimated by Student's *t*-test or nonparametric Mann–Whitney *U* test. Chi square test or Fisher exact test was used for assessing differences in frequencies between groups. The level of significance was set at *p* < 0.05.

## 3. Results

### 3.1. Prevalence of Autoantibodies

Among 128 patients with T2D, 13 cases (10.16%) had positive autoantibodies. Twelve (92.3%) were positive for GAD65A only, one (7.7%) for IA-2A only, and none for ZnT8A. For all patients with previously diagnosed autoimmune diabetes, the presence of autoantibodies was confirmed—all (100%) were positive for GAD65A and 6 (42.9%) were also positive for IA-2A. Only one of the assayed LADA patients (*n* = 7) was positive for ZnT8A with 255.73 IU/ml, and in this case, all analyzed autoantibodies were positive (GAD65A-248.7 IU/ml; IA-2A-2356 IU/ml). None of the control subjects (*n* = 38) was positive for GAD65A and IA-2A. The level of ZnT8A was above the reference range in one control participant (22 IU/ml). Levels of GAD65A in the newly found cases of positive autoantibodies differed significantly in comparison with those in LADA (Table 1). The level of IA-2A varied widely from 26 IU/ml to 2356 IU/ml.

### 3.2. Metabolic Features

Differences between age of patients with T2D, autoantibody-negative and autoantibody-positive cases of the group, and LADA participants were not significant. Neither the age at diagnosis and duration of the disease was significantly different (Table 2).

Significance in gender distribution between groups (autoantibody-negative—55.7 versus 44.3%, newly found autoantibody-positive—46.2 versus 53.8%, LADA—78.6 versus 21.4%, and control subjects—52.6 versus 47.4% for female versus male, resp.) was excluded (Chi square = 3.588, *p* = 0.310, phi = 0.141).

Preliminary comparative analysis between patients enrolled with T2D and control subjects showed significance concerning difference in BMI and waist circumference, systolic and diastolic blood pressure, and FPG and triglycerides' level (*p* < 0.001). Results were confirmed after exclusion of cases with positive autoantibodies (*p* < 0.001) (Table 3).

Analysis between autoantibody-negative and autoantibody-positive cases within the T2D group demonstrated lower BMI (*p* = 0.014), but not significantly lower waist circumference, worse glycemic control (HbA1c, *p* = 0.033; PPG, *p* = 0.003), but better HDL cholesterol (*p* = 0.026) in the latter (Table 3).

Analysis between patients enrolled in LADA group and cases who were found to have positive autoantibodies within the T2D group showed significant difference in systolic (*p* = 0.013) and diastolic blood pressure (*p* = 0.012), and VLDL level (*p* = 0.035). Anthropometric data, glycemic control, and the rest of the lipid profile parameters were similar (nonsignificant *p* value) (Table 3).

Comparison between autoantibody-negative patients and those enrolled in LADA cohort showed a significant difference for HbA1c (*p* = 0.001), anthropometric data, arterial blood pressure, and lipid profile (*p* < 0.001) (Table 3).

Metabolic syndrome characteristics were strongly presented in subjects with T2D who proved negative for diabetes-associated autoantibodies. All of its features except glycemia were milder in participants with positive autoantibodies (Table 3). Prevalence of metabolic syndrome, diagnosed according to the cited NCEP criteria, was significantly lower among patients from the T2D group who eventually had positive autoantibodies when compared to the autoantibody-negative cases but remained the lowest among participants with LADA (Table 4).

Twenty percent (*n* = 23) of antibody-negative T2D patients were receiving insulin therapy versus 69.2% (*n* = 9) of those in T2D group who had positive antibodies (*p* < 0.001). Duration of diabetes before initiation of insulin was longer in the first cohort (2.93 ± 2.74 years) compared to both newly found cases with positive antibodies (1.57 ± 1.81 years) and subjects enrolled in LADA group (1.82 ± 1.51 years), but these differences were not significant.

### 3.3. Inflammatory Cytokines

Comparison between autoantibody-negative T2D participants and control subjects showed significance regarding levels of hs-CRP and IL-6 (*p* < 0.001). Some markers of both cohorts with positive autoantibodies—the newly diagnosed (IL-6, *p* < 0.001) and LADA (hs-CRP, *p* = 0.046), differed from those in the control group too.

The level of the inflammatory markers of the cases with positive autoantibodies within the T2D group was not much altered compared to those who were autoantibody-negative (nonsignificant *p* value), and even IL-6 was significantly higher than that in the enrolled as LADA cases (*p* = 0.002). The difference in the level of TNF*α* was not significant between any of the groups (Table 3).

## 4. Discussion

According to the present study, the prevalence of positive diabetes-associated autoantibodies among patients diagnosed with T2D is 10.16%, which is within the range cited in studies in European countries [9].

GAD65As were the main positive autoantibody and followed by IA-2A. Assaying both GAD65A and IA-2A but not ZnT8A contributed to finding new cases of positive diabetes-associated autoantibodies. The number of positive autoantibodies but not GAD65A titer has been considered a major risk factor for disease progression in LADA [22]. However, in the current study, LADA patients demonstrated a significantly higher level of GAD65A compared to subjects who were enrolled in T2D group. Besides, 42.9% of subjects in LADA group were positive for more than one autoantibody compared to none in the T2D cohort. The number and titer of autoantibodies have been used for classifying two types of LADA—one with the clinical features of T1D (LADA-type 1) and another with a clinical and metabolic phenotype of T2D (LADA-type 2) [23]. Despite the facts that we have not considered a comparison to T1D and that anthropometric and metabolic data still differed significantly between participants from T2D group with positive and negative autoantibodies, both cohorts of cases with positive autoantibodies were not the same.

Analysis of the criteria used in the assessment of metabolic syndrome unequivocally showed that it was more prevalent in patients with T2D compared to those with adult-onset autoimmune diabetes. A significantly higher incidence of the syndrome in patients from T2D group with newly found positive autoantibodies was also observed when compared to that in patients who were already diagnosed with LADA. However, the blood pressure control was the only criterion that demonstrated a significant difference between both cohorts as they shared similar glycemic and lipid control and waist circumference measurements.

It is well known that T2D and metabolic syndrome are associated with a chronic low-grade inflammation [24]. Indeed, patients with both T2D and LADA had significantly altered IL-6 and hs-CRP levels compared to control subjects. This difference was significant between both types of diabetes too. Comparative analysis between participants in the T2D group who proved negative and those who were positive for the analyzed autoantibodies showed no significance concerning IL-6 and hs-CRP. In contrast, such was present between both cohorts with positive autoantibodies and this result was independent of anthropometric data and glycemic control as the difference between them for these two groups was not significant.

In this way, patients with positive antibodies enrolled as diagnosed with T2D shared common features with both classical T2D and adult-onset autoimmune diabetes.

The current study has some limitation factors. The main one is the low number of patients. Specificity for both anti-GAD65 and anti-IA2 ELISA kits was high at 98 and 99%, respectively, but still allows for the presence of false positive results. The difference in the results of the analyzed metabolic and inflammatory characteristics of patients who had positive autoantibodies within the T2D group, those who were negative, and subjects enrolled in the LADA group could be influenced by a false positive rate within the T2D cohort. This could be a possible reason why subjects with positive antibodies from T2D group had similar glycemic and anthropometric parameters with participants from LADA cohort but shared common inflammatory status with patients who proved antibody-negative. Despite that, the percentage of patients diagnosed with T2D who have positive antibodies is not to be neglected.

Antibody titer could vary and even become within reference range at some point in time suggesting a previously false positive result [9]. Having a cross-sectional design, this study only reflects the current status of diabetes-associated antibodies. It demonstrates that subjects who have not been considered for the analysis of antibody status in ambulatory practice are more likely to have a lower titer and be positive for only one antibody, unlike patients with the typical phenotype of autoimmune diabetes. Follow-up would reveal whether antibodies would remain high or the currently measured level would be rendered false positive. Despite titer fluctuations and results with borderline values, transitional outreaching of reference range might suggest a predisposition to autoimmune response and autoreactive T cells have been described in autoantibody-negative patients [25].

Patients with LADA usually have autoantibodies assayed because of unsatisfying and unstable glycemic control, low BMI, family history, or concomitant autoimmune diseases. Glycemic control is a chief goal in the treatment of diabetes mellitus. The presence of autoantibodies, as also seen from our results, is associated with greater efforts in achieving optimal glycemic targets, and this has been related to a lower residual beta-cell function than in autoantibody-negative cases [26].

It is still not known which is the best treatment aimed to preserve *β*-cell function in autoimmune diabetes and whether early insulin initiation will ensure this [27–29]. When it comes to patients thought to have T2D, insulin treatment is often postponed despite worsening HbA1c. Reasons include weight gain, hypoglycemia risks, patients' reluctance to undertake this treatment, and so forth. Routine assaying for autoantibodies could diagnose autoimmune diabetes among T2D even when LADA is less suspected. Awareness of autoimmunity will result in timely initiation of insulin in these cases specifically and will not only prevent acute metabolic decompensation but also impact development of chronic vascular complications.

## 5. Conclusion

The current study showed 10% prevalance of positive diabetes-associated autoantibodies among T2D in the analyzed sample of the Bulgarian population. Patients with newly found positive diabetes-associated antibodies shared common metabolic and anthropometric features with subjects with LADA, but analyzed inflammatory markers were closer to those of participants with classical T2D.

## Figures and Tables

**Table 1:** Level of GAD65A in autoantibody-positive diabetic patients.

	GAD65A (IU/ml) in LADA (*n* = 14)	GAD65A (IU/ml) in Ab^+^T2D (*n* = 12)	*p*
М (SD)	192.94 (78.77)	109.06 (75.61)	0.014
Md	232.30	113.10	
Minimum	25.80	11.80	
Maximum	274.90	210.80	

M: mean; SD: standard deviation; Md: median; *n*: number; GAD65A: antiglutamic acid decarboxylase 65 autoantibodies; LADA: latent autoimmune diabetes of the adults; Ab^+^T2D: autoantibody-positive cases in T2D group.

**Table 2:** Age, duration of diabetes, and age at diagnosis of diabetic patients and control subjects.

	T2D (*n* = 128)	Ab^−^T2D (*n* = 115)	Ab^+^T2D (*n* = 13)	LADA (*n* = 14)	Controls (*n* = 38)
Age (y)	53.83 (6.76)^∗^	53.76 (6.66)^∗^	54.46 (7.86)^∗^	50.07 (7.86)	52.34 (4.17)
Age at diagnosis (y)	49.51 (7.53)	49.37 (7.42)	50.69 (8.68)	45.88 (8.44)	—
Diabetes duration (y)	4.36 (3.14)	4.43 (3.21)	3.77 (2.39)	3.27 (2.51)	—

Results are expressed as mean (standard deviation); *n*: number; T2D: type 2 diabetes; LADA: latent autoimmune diabetes of the adults; Ab-T2D: autoantibody-negative cases in T2D group; Ab^+^T2D: autoantibody-positive cases in T2D group; ^∗^*p* < 0.05 compared with control group only.

**Table 3:** Anthropometric data, blood pressure, and metabolic control of subjects.

	T2D (*n* = 128)	Ab^−^T2D (*n* = 115)	Ab^+^T2D (*n* = 13)	LADA (*n* = 14)	Controls (*n* = 38)
BMI (kg/m^2^)	32.70 (6.58)^∗∗^	33.18 (6.37)^∗∗^	28.49 (7.13)^†^^∗^	24.94 (3.54)^‡^	23.68 (3.34)
WC (cm)	103.67 (14.49)^∗∗^	104.40 (14.04)^∗∗^	96.83 (17.35)	85.43 (9.32)^‡^	88.29 (13.18)
SBP (mmHg)	135.82 (19.17)^∗∗^	136.78 (19.49)^∗∗^	127.31 (13.94)	112.86 (12.51)^‡^	119.00 (16.08)
DBP (mmHg)	83.91 (9.80)^∗∗^	84.00 (9.96)^∗∗^	83.08 (8.55)^∗^	73.21 (9.12)^‡^	74.57 (10.10)
HbA1c (%)	7.97 (1.86)	7.87 (1.86)	8.89 (1.67)^†^	9.40 (1.16)^‡^	—
FPG (mmol/l)	7.97 (2.94)^∗∗^	7.81 (2.72)^∗∗^	9.45 (4.24)^∗∗^	7.79 (2.62)^∗^	5.28 (0.47)
PPG (mmol/l)	8.35 (3.26)	8.05 (3.10)	11.26 (3.49)^‡^	9.53 (4.19)	—
TC (mmol/l)	5.22 (1.15)	5.17 (1.11)	5.70 (1.42)	5.01 (1.11)	5.28 (0.90)
HDL (mmol/l)	1.29 (0.39)	1.25 (0.35)	1.69 (0.55)^†^^∗^	1.76 (0.49)^‡^^∗^	1.30 (0.55)
LDL (mmol/l)	3.03 (0.96)	3.02 (0.90)	3.10 (1.45)	2.39 (1.21)	3.28 (1.02)
VLDL (mmol/l)	0.83 (0.36)	0.83 (0.34)	0.79 (0.53)	0.40 (0.11)^‡^	0.72 (0.50)
TG (mmol/l)	1.90 (0.99)^∗∗^	1.91 (0.97)^∗∗^	1.78 (1.17)	1.08 (0.97)^‡^	1.33 (1.17)
Hs-CRP (mg/l)	2.40 (1.61)^∗∗^	2.50 (1.60)^∗∗^	1.61 (1.52)	1.44 (1.39)^†^^∗^	0.92 (1.27)
IL-6 (pg/ml)	3.85 (4.76)^∗∗^	3.90 (4.88)^∗∗^	3.45 (3.58)^∗∗^	1.40 (0.00)^‡^	1.43 (0.18)
TNF*α* (pg/ml)	14.57 (16.70)	15.45 (17.38)	7.71 (7.83)	13.47 (17.96)	16.22 (18.14)

Results are expressed as mean (standard deviation); *n*: number; T2D: type 2 diabetes; LADA: latent autoimmune diabetes of the adults; Ab^−^T2D: autoantibody-negative cases in T2D group; Ab^+^T2D: autoantibody-positive cases in T2D group; BMI: body mass index; WC: waist circumference; DBP: systolic blood pressure; DBP: diastolic blood pressure; FPG: fasting plasma glucose; PPG: postprandial plasma glucose; TC: total cholesterol; HDL: high-density lipoprotein; LDL: low-density lipoprotein; VLDL: very low-density lipoprotein; TG: triglycerides; hs-CRP: high sensitivity C-reactive protein; IL-6: interleukin 6; TNF*α*: tumor necrosis factor alpha; ^∗^*p* < 0.05 compared to control group, ^∗∗^*p* < 0.005 compared to control group, ^†^*p* < 0.05 compared to Ab^−^T2D group, ^‡^*p* < 0.005 compared to Ab^−^T2D group.

**Table 4:** Prevalence of metabolic syndrome in T2D and LADA.

	Ab^−^T2D	Ab^+^T2D	LADA
No metabolic syndrome *N* (%)	12 (10.4)	6 (46.2)	12 (85.7)
With metabolic syndrome *N* (%)	103 (89.6)	7 (53.8)	2 (14.3)
		*p* = 0.003^∗^	*p* < 0.001^∗^
		*p* = 0.046^∗∗^	

T2D: type 2 diabetes; LADA: latent autoimmune diabetes of the adults; Ab^−^T2D: autoantibody-negative cases in T2D group; Ab^+^T2D: autoantibody-positive cases in T2D group; *N*: number; ^∗^*p* value compared to Ab^−^T2D, ^∗∗^*p* value between Ab^+^ T2D and cases of LADA.
